# ABCC6 Transporter Contributed to Cisplatin Resistance on Bladder Cancer

**DOI:** 10.7150/ijms.115487

**Published:** 2025-08-22

**Authors:** Kuang‑Yu Chou, An-Chen Chang, Thomas I‑Sheng Hwang, Te‑Fu Tsai, Chao‑Yen Ho, Hsiu-Wen Liu, David Achudhan, Chih-Hsin Tang, Yen-You Lin

**Affiliations:** 1 Division of Urology, Department of Surgery, Shin Kong Wu Ho‑Su Memorial Hospital, Taipei 111045, Taiwan.; 2 School of Oral Hygiene, College of Oral Medicine, Taipei Medical University, Taipei 11031, Taiwan.; 3 Translational Medicine Center, Research Department, Shin Kong Wu Ho‑Su Memorial Hospital, Taipei 111045, Taiwan.; 4 Division of Urology, School of Medicine, Fu‑Jen Catholic University, New Taipei 242062, Taiwan.; 5 Department of Urology, Taipei Medical University, Taipei 11031, Taiwan.; 6 Institute of Traditional Medicine, School of Medicine, National Yang Ming Chiao Tung University, Taipei 112304, Taiwan.; 7 Department of Physiology Biomedical Engineering, Mayo Clinic, Rochester, Minnesota 55905, USA.; 8 Department of Pharmacology, School of Medicine, China Medical University, Taichung404328, Taiwan.; 9 Graduate Institute of Biomedical Sciences, China Medical University, Taichung404328, Taiwan.; 10 Chinese Medicine Research Center, China Medical University, Taichung404328, Taiwan.; 11 Department of Medical Laboratory Science and Biotechnology, Asia University, Taichung413305, Taiwan.

**Keywords:** bladder cancer, cisplatin resistance, ABC transporter C member 6 transporter, autophagy, chemotherapy resistance

## Abstract

Bladder cancer (BLCA) is one of the most common urological malignancies worldwide, including in Taiwan, where its incidence has been increasing. It accounts for approximately 3% of all newly diagnosed cancer cases. Drug resistance remains a major challenge in BLCA treatment, particularly at the metastatic stage, where only 35% of metastatic BLCA patients respond to cisplatin chemotherapy, and most eventually develop resistance. In the present study, we established cisplatin-resistant BLCA cell models (T24 and UMUC3) and analyzed ATP-binding cassette (ABC) transporters. We identified ABC transporter C member 6 (ABCC6) as a crucial transporter upregulated in cisplatin-resistant BLCA cells. Further analysis showed that ABCC6 knockdown affected autophagy-related markers, and inhibition of autophagy using bafilomycin A1 (BafA1) and chloroquine (CQ) reversed cisplatin resistance. These findings suggest that ABCC6 contributes to cisplatin resistance in BLCA through autophagy regulation. Our study highlights ABCC6 as a crucial factor in BLCA cisplatin resistance, with autophagy playing a significant role in this mechanism. Targeting autophagy may offer a potential strategy to overcome chemoresistance in BLCA. Future research should focus on validating these findings in clinical samples and exploring ABCC6 inhibitors or autophagy modulators as therapeutic options.

## Introduction

Bladder cancer (BLCA) is a common malignant tumor of the urinary system that originates in the tissues of the bladder, the organ responsible for urine storage. Globally, BLCA ranks 13^th^ in terms of mortality and 10^th^ in terms of frequency of diagnosis 1. The Global Cancer Incidence, Mortality, and Prevalence (GLOBOCAN) data indicate that there were approximately 573,000 instances of BLCA detected in 2020, making up around 3% of all new cancer cases diagnosed 1. GLOBOCAN 2022 further reported that bladder cancer was responsible for approximately 614,298 new cases worldwide, representing a 7.1% increase compared to 2020. In total, there were an estimated 19.9 million cancer cases and 9.7 million cancer-related deaths globally 2. BLCA predominantly affects older adults, with key risk factors including smoking, exposure to certain chemicals such as aromatic amines, and dyes etc., and persistent bladder inflammation 3. It is often detected through symptoms such as visible or microscopic blood in the urine (hematuria), increased urinary frequency, and pelvic discomfort 4. For clinical treatment, radical cystectomy is still the gold standard of care for Muscle-Invasive BLCA; however, because of advanced age, poor performance status, or many comorbidities, some patients are not candidates for curative surgery. Furthermore, severe surgery and urine diversion have a major negative impact on the quality of patients' life 5. Other treatments for BLCA include immunotherapy, photodynamic therapy, radiation, and intravesical chemotherapy.

Among platinum-based anti-neoplastic agents, cisplatin is the first and most effective 6. It is used to treat a variety of solid tumors, such as lung, ovarian, cervical, and BLCA 7. Since the late 1980s, cisplatin-based combination chemotherapies have been utilized as significant adjuvant therapy for patients with metastatic BLCA 8. Reducing the side effects while maintaining the anticancer capabilities of cisplatin led to the development of several additional platinum compounds, such as carboplatin, oxaliplatin, and picoplatin 9,10. Nevertheless, cisplatin remains a crucial component of BLCA treatment. Unfortunately, only 35% of patients with metastatic BLCA initially respond to cisplatin-based chemotherapy, and most patients with BLCA who do respond to the treatment later become resistant to it 8,11,12. Patients with metastatic BLCA have a dismal prognosis, in part because of resistance to cisplatin. Thus, drug resistance continues to be a key barrier to the development of successful therapeutic interventions against BLCA, despite tremendous advancements in research and the creation of cancer treatment tactics like targeted therapy and immunotherapy.

The well-known drug efflux transporters, such as P-glycoprotein (P-gp), play a crucial role in chemotherapy failure by actively expelling anticancer drugs from cells. Among them, ATP-binding cassette (ABC) transporters constitute a large family of membrane proteins that utilize energy from ATP hydrolysis to transport a wide variety of substrates across cellular membranes 13-15. ABC transporters also contribute significantly to cisplatin resistance by decreasing intracellular drug accumulation, thereby reducing its cytotoxic effects. Although cisplatin is not a classical substrate of ABC transporters, certain multidrug resistance-associated proteins (MRPs, ABCC family) - such as MRP2 (ABCC2) and MRP4 (ABCC4) - facilitate the efflux of its metabolites (e.g. glutathione-conjugated cisplatin), lowering the intracellular concentration of the active drug 16. Additionally, studies suggest that the breast cancer resistance protein (BCRP, ABCG2) may be involved in cisplatin resistance by influencing drug accumulation in tumor cells 17. Cisplatin primarily enters cells through the copper transporter CTR1 (SLC31A1). However, the overexpression of certain ABC transporters, such as P-glycoprotein (ABCB1), may indirectly downregulate CTR1 expression, leading to reduced cisplatin uptake 18. Overall, ABC transporters contribute to cisplatin resistance through a combination of drug efflux, reduced drug uptake, and enhanced detoxification mechanisms. Given these challenges, this study aims to explore strategies to enhance clinical efficacy in resistant cancers by targeting ABC transporter-mediated resistance.

## Materials and Methods

### Materials

ATG5 (sc-133158), ATG12 (sc-271688), ABCC6 (sc-59618), p62 (sc-48402) and b-actin (sc-58673) antibody were bought from Santa Cruz Biotechnology, Inc. (Santa Cruz, CA, USA). Cell culture materials were bought from Thermo Fisher Scientific, Inc. (Waltham, MA, USA). The qPCR primers and ABCC6 short hairpin (sh) RNA were purchased from MDBio, Inc (New Taipei, Taiwan). The Taqman^®^ one-step PCR Master Mix were supplied by Applied Biosystems (Foster City, CA, USA). Pharmacological inhibitors for autophagy bafilomycin A1 (BafA1) and chloroquine (CQ) were supplied by Sigma-Aldrich (St. Louis, MO, USA). Other chemicals not mentioned above were purchased from Sigma-Aldrich (St. Louis, MO, USA).

### Cell culture

The human BLCA cell lines T24 (grade III) and UMUC3 (stage III) were obtained from the Bioresource Collection and Research Center (BCRC) in Taiwan. T24 cells were cultured in McCoy's 5A medium (Gibco; Thermo Fisher Scientific, Inc.), whereas UMUC3 cells were cultured in Minimum Essential Medium (Gibco). Both media contained 10% fetal bovine serum (FBS; Gibco), 2 mM GlutaMAX‑1, and Penicillin/Streptomycin/Amphotericin B Solution (Sigma‑Aldrich; Merck KGaA). The cells were incubated at 37°C with 5% CO₂.

### Cisplatin-resistant cell establishment

Cisplatin-resistant BLCA cell lines were established from T24 and UMUC3 cells following a previously described protocol 19. Each cell line was subjected to increasing concentrations of cisplatin continuously for a duration of six months. And the IC_50_ were subsequently determined for cisplatin resistance T24 (T24R) cell and cisplatin resistance UMUC3 (UMUC3R) cell. Wild-type cells cultured on the culture medium without cisplatin resistance used as a control.

### Cell viability

T24 and UMUC3 cells were seeded in 96‑well plates at a density of 2×10⁴ cells per well. The cells were then treated with varying concentrations of cisplatin (0, 3.125, 6.25, 12.5, 25, 50, 100, 150, and 200 μM) for 24 h. Cell viability was evaluated using a resazurin reagent (Biotium, Inc.). And resazurin solution, constituting 10% of the initial well volume, was added, and the plate was incubated for 6 h at 37°C under 5% CO₂. Fluorescence intensity was subsequently measured using a multimode microplate reader (Varioskan LUX Plate Reader; Thermo Fisher Scientific, Inc.).

### shRNA Transfection

Transfection of 1 μg ABCC6 shRNA plasmid into BLCA cells was carried out using the ViaFect™ transfection reagent (Promega, WI, USA), following the manufacturer's protocol.

### Colony formation

T24 and UMUC3 cells were seeded into 6‑well plates at a density of 3×10³ cells per well and treated with varying concentrations of cisplatin (0, 0.5, 1, 2, or 6 μM). After a 7-day incubation period, colonies containing ≤50 cells were identified. The cell colonies were fixed with 3.7% formaldehyde for 20 min at room temperature (RT), followed by staining with 0.05% crystal violet (w/v) for another 20 mins at RT. The stain was then extracted using 10% acetic acid, and the absorbance of the resulting solution was measured to quantify the cell colonies 20.

### Western blotting

Protein samples were resolved on sodium dodecyl sulfate polyacrylamide gel electrophoresis and transferred to immobilon polyvinylidene difluoride membranes. Membranes were then blocked with protein‑free blocking buffer (Thermo Fisher Scientific, MA, USA) for 1 h at RT, followed by incubation with primary antibodies against p62, LC3-II, ATG5, ATG12, and β‑actin (1:3000; GeneTex, Irvine, CA, USA) overnight at 4°C. After three washes with PBST, membranes were subsequently incubated with peroxidase‑conjugated secondary antibodies (1:3000; GeneTex, Irvine, CA, USA) for 1 h at RT. Protein bands were visualized with enhanced chemiluminescence using Kodak X‑OMAT LS film (Eastman Kodak, Rochester, NY, USA).

### Real-time polymerase chain reaction (qPCR)

Total RNA was isolated using the easy‑BLUE™ Total RNA Extraction Kit (iNtRON biotechnology Inc. WA, USA). mRNA was subsequently reverse-transcribed into cDNA utilizing the MMLV Reverse Transcriptase kit (Invitrogen; Thermo Fisher Scientific, Inc. MA, USA) and the Mir-X™ miRNA First-Strand Synthesis kit (Takara Bio, Kyoto, Japan) in accordance with the manufacturer's instructions. qPCR analysis was carried out using the SYBR Green Master Mix (Applied Biosystems; Thermo Fisher Scientific, Inc. MA, USA) on a StepOnePlus sequence detection system (Thermo Fisher Scientific, Inc. MA, USA). The thermal cycling conditions were consistent with those described in previous studies 20,21. qPCR primers used for mRNA detection are detailed in Table 1. Relative gene expression levels were calculated using the comparative Ct (2^‑ΔΔ^Ct) method, with gene expression normalized to GAPDH.

### Calcein AM assay

T24 and UMUC3 cells were seeded into 48‑well plates at a density of 1×10⁴ cells per well and treated according to the experimental conditions. The cells were pre‑stained with calcein AM, (a green, fluorescent dye; 1 μg/μl), for 1 hour at 37°C in an incubator, followed by three washes with phosphate‑buffered saline (PBS). Subsequently, the cells were incubated with calcein AM staining reagent at a ratio of 1:5 for 4 h at 37°C. Viable BLCA cells were identified based on their green fluorescence signal, which was measured using a Varioskan LUX Plate Reader (Thermo Fisher Scientific, Inc. MA, USA) at the corresponding fluorescence wavelength.

### Fluorescent staining

T24 and UMUC3 cells were placed onto chamber slides (Sigma‑Aldrich; Merck KGaA, MA, USA) and treated according to the experimental conditions. The cells were incubated with primary antibodies targeting LC3-II (1:50 dilution; Cell Signaling Technology, Inc. MA, USA) and p62 (1:50 dilution; Cell Signaling Technology, Inc. MA, USA) for 1 h at RT, followed by counterstaining with 4',6‑diamidino‑2‑phenylindole (DAPI) for 5 min at RT. LC3-II and p62-positive BLCA cells were visualized using a Nikon Ti2 fluorescence microscope (Nikon Corporation, Tokyo, Japan).

### Statistical analysis

All experiments were conducted three times independently. Comparisons between two groups were analyzed using the student's *t-test*, while one-way ANOVA was employed for comparisons involving three or more groups. Results are expressed as mean values with standard deviations (mean ± SD). The *p* value of less than 0.05 was considered to indicate statistical significance.

## Results

### Effects of cisplatin resistance on BLCA

**Figure 1:**
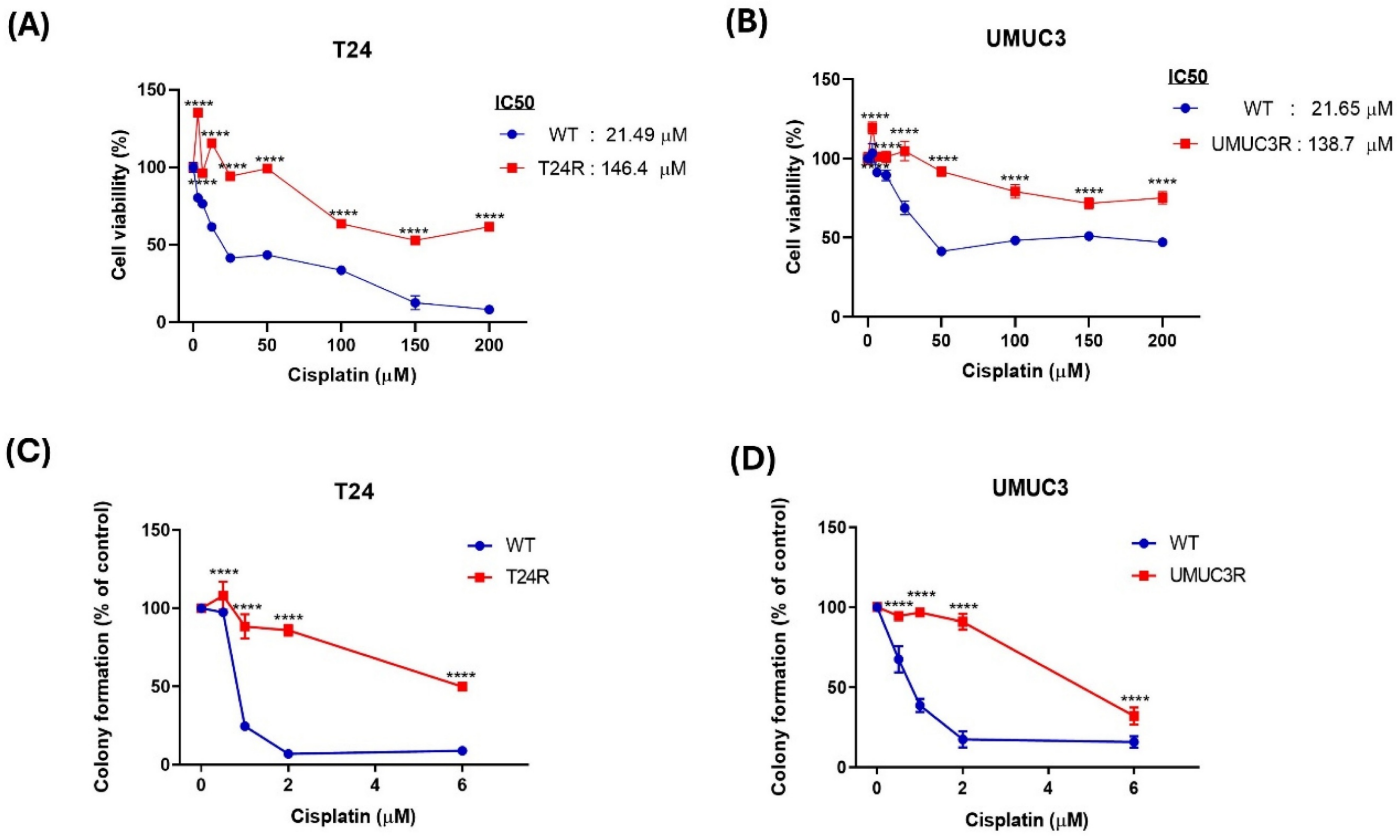
** Comparative Analysis of Cisplatin Resistance in T24 and UMUC3 BLCA Cells. (A&B)** Cell viability of T24 and UMUC3 cells treated with varying concentrations of cisplatin (0 to 200 µM). **(C&D)** Colony formation assay for T24 and UMUC3 cells showing cisplatin resistance. T24R: T24 cell with cisplatin resistance; UMUC3R: UMUC3 cell with cisplatin resistance. *p> 0.05 vs. WT group.

To investigate molecular changes associated with cisplatin resistance in BLCA, a resistance model was established by subjecting wild-type cells to incremental doses of cisplatin. A cell viability assay was performed to determine the IC_50_ values by exposing the cells to increasing concentrations of cisplatin. The IC_50_ value for wild-type T24 cells was found to be 21.49 μM, while that for wild-type UMUC3 cells was 21.65 μM. Further, the long-term survival ability of the model was assessed, and the results show that the cisplatin resistant T24 and UMUC3 cells exhibited significantly higher IC₅₀ values than the wild-type cells which the IC_50_ value for the T24R cell was 146.4 mM and the UMUC3R was 138.7 mM. (Fig. 1A &B). For cell proliferation, colony formation assays were conducted. And the colony formation assay also shows similar results that the cisplatin resistant T24 and UMUC3 cells have higher colony formation than wild-type cells (Fig. 1C &D). Based on these results, the cisplatin resistance in BLAC is well-established in this study.

### Effects of cisplatin resistance on ABCs transporter expression

**Figure 2:**
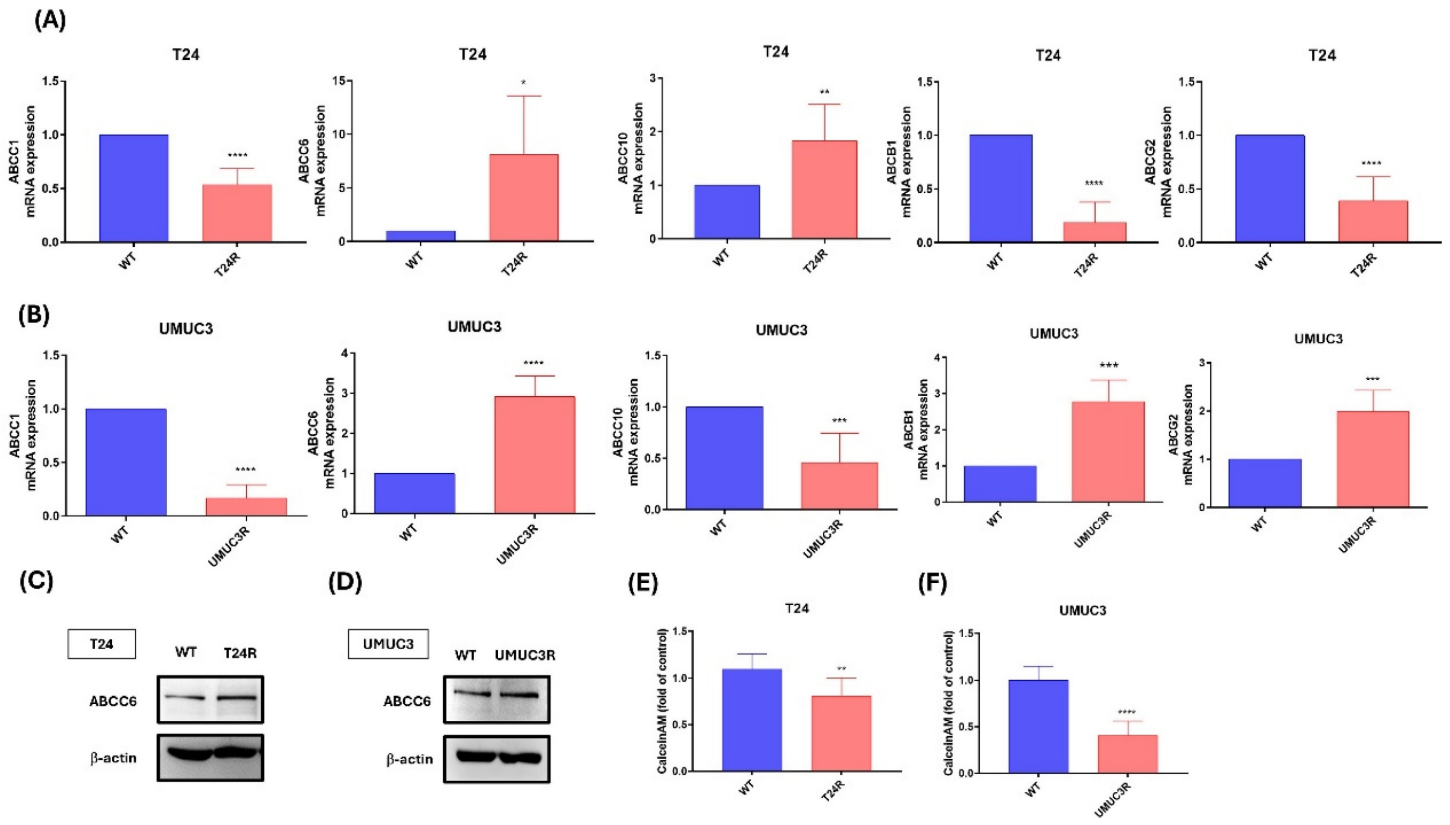
** The Role of ABC Transporters in Cisplatin Resistance of T24 and UMUC3 BLCA Cells. (A)** mRNA expression levels of ABC transporters in T24 cells. **(B)** mRNA expression levels of ABC transporters in UMUC3 cells, **(C)** Protein levels of ABCC6 in T24 cells. **(D)** Protein levels of ABCC6 in UMUC3 cells. **(E)** Calcein AM efflux in T24 cells: **(F)** Calcein AM efflux in UMUC3 cells. * p <0.05, **p <0.01, *** p <0.001 and **** p <0.0001, vs. WT group.

To investigate the effect of cisplatin resistance, qPCR was used to analyze the different types of ABCs transporter at mRNA levels. In Fig. 2A, the results show that cisplatin resistance is reducing ABCC1, ABCB1 and ABCG2 mRNA expression and increasing ABCC6 and ABCC10 mRNA expression on T24 cell between cisplatin resistance and wild type. In Fig. 2B, the results show that cisplatin resistance reduces ABCC1, and ABCC10 mRNA expression and increasing ABCC6, ABCB1 and ABCG2 mRNA expression on UMUC3 cell between cisplatin resistance and wild type. Thus, we based on the above results, ABCC6 emerged as a consistently upregulated transporter which cisplatin inducing drugs resistance on BLCA. For further evidence, western blotting analysis was used, and the blotting images also show that higher protein expression on cisplatin resistance compared to wild type cells (Fig. 2C). And the calcein AM staining also was used for the transporter function. The result shows the lower calcein AM staining fold on T24R and UMUC3R cells with comparing to wild type (Fig. 2D).

### Effects of ABCC6 blocking reducing cisplatin resistance

**Figure 3:**
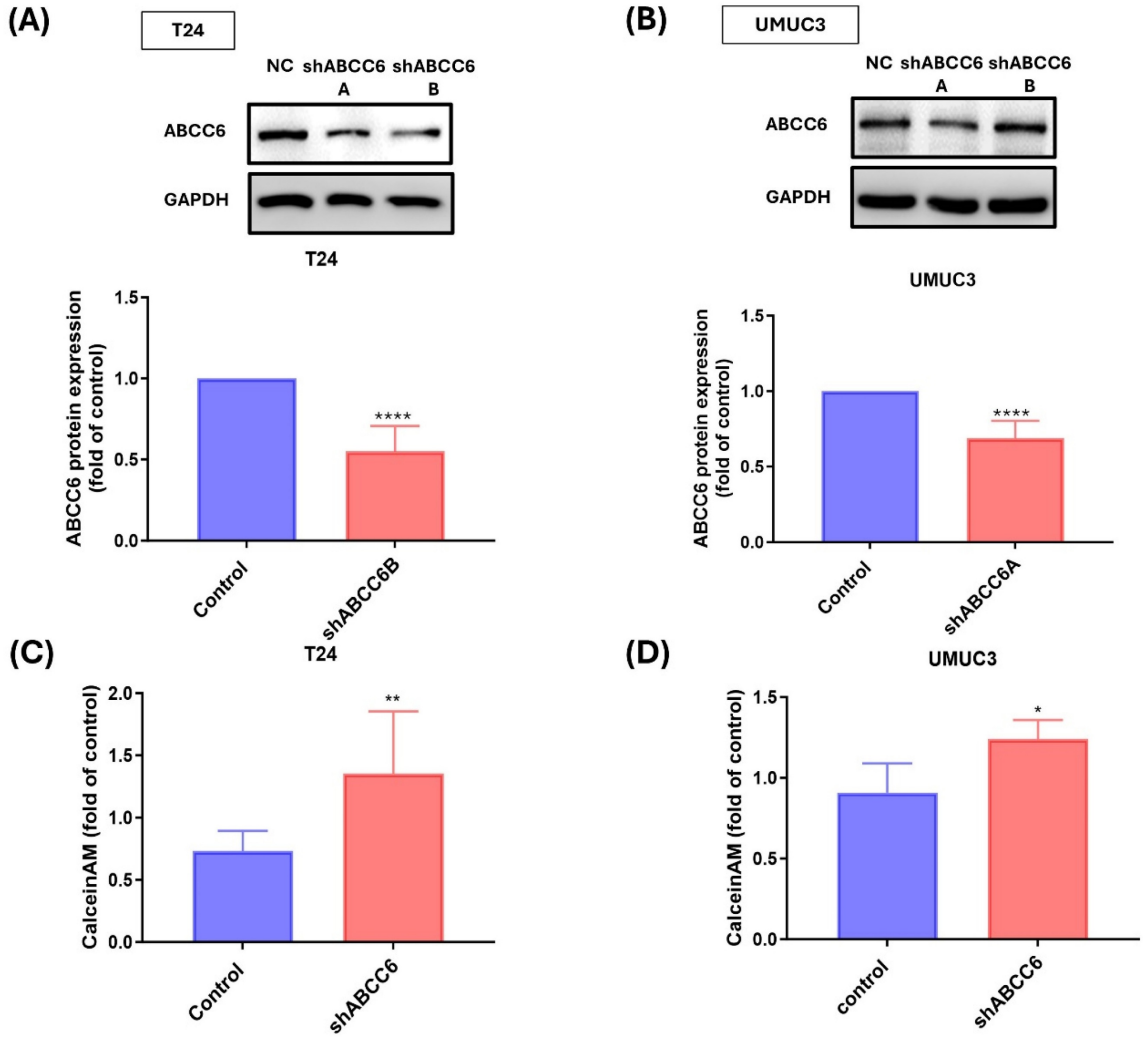
** The Role of ABCC6 in Cisplatin Resistance of T24 and UMUC3 BLCA Cells. (A)** Western blot analysis showing ABCC6 protein levels in T24 cells under wild-type and cisplatin-resistant conditions. **(B)** Western blot analysis illustrating ABCC6 protein levels in UMUC3 cells under WT and cisplatin-resistant conditions. **(C)** Bar graph representing the fold changes in calcein AM efflux in T24 cells, comparing control and shABCC6-treated. **(D)** Bar graph illustrating the fold change in calcein AM efflux in UMUC3 cells, comparing control and shABCC6-treated conditions. * p <0.05, ** p <0.01, ***p<0.001 and **** p <0.0001, vs. control group.

To confirm the role of ABCC6 on cisplatin resistance BLCA cell, two short-hairpin (sh) RNAs of ABCC6 were used for inhibiting ABCC6 expression. The results show that the two ABCC6 shRNA effectively reduced ABCC6 protein expression either on cisplatin resistance T24 cell or cisplatin resistance UMUC3 cell (Fig. 3A&B). And the ABCC6 mRNA expression also was inhibited by ABCC6 shRNA transfection on BLCA cell line. And the calcein AM staining also was used to evaluate the effects of ABCC6 shRNA on BLCA. And the results show the higher calcein AM staining fold on T24R and UMUC3R cells compared to control cells (Fig. 3C&D).

### Effects of cisplatin resistance on Autophagy of BLCA

**Figure 4:**
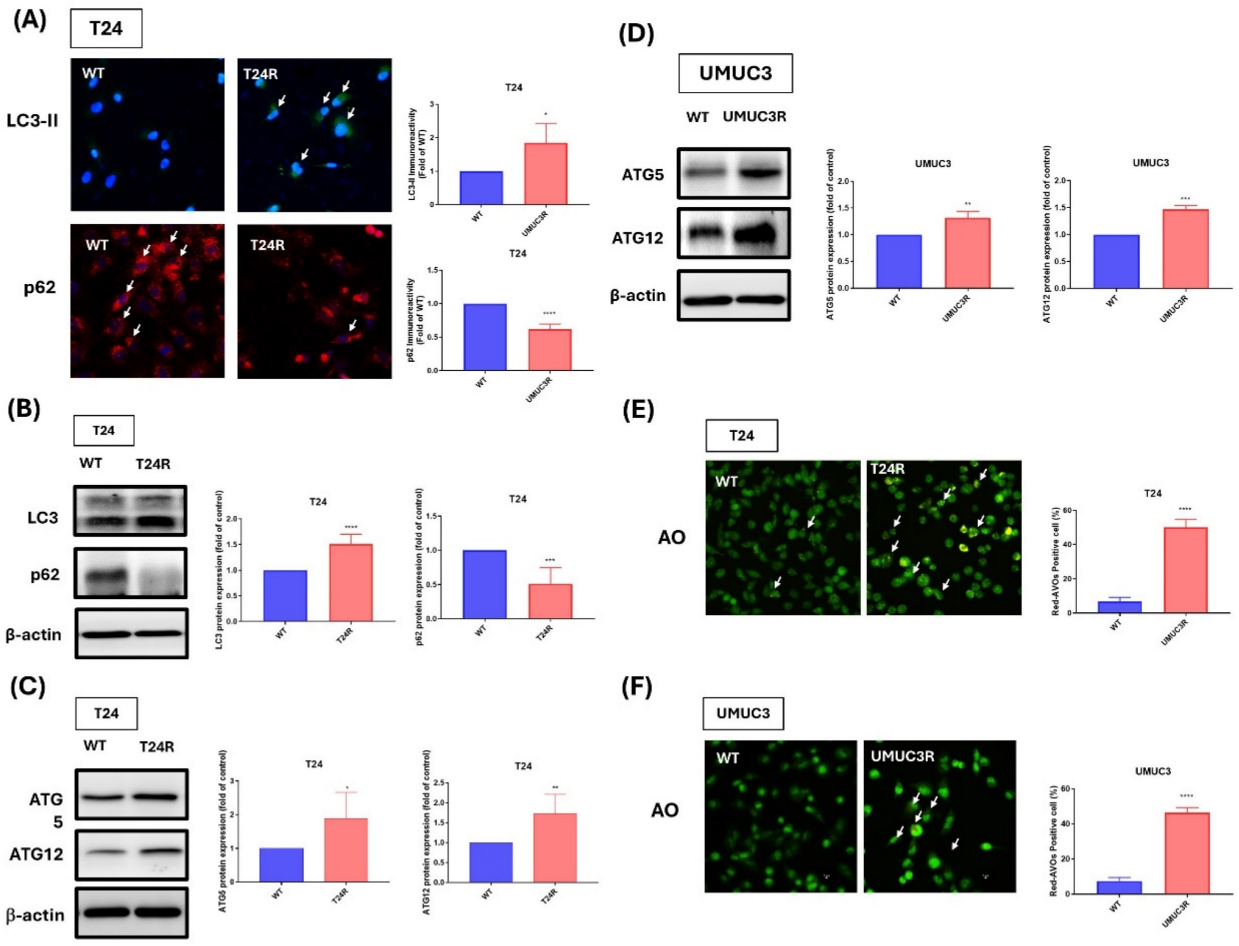
** Comparative Analysis of Autophagy and Protein Expression in Cisplatin-Resistant T24 and UMUC3 BLCA Cells. (A)** Immunofluorescence staining of LC3-II and p62 in T24 cells **(B)** Western blot analysis of LC3 and p62 in T24 cells. **(C)** Western blot analysis of ATG5 and ATG12 in UMUC3 cells. **(D)** Western blot analysis of ATG5 and ATG12 in T24 cells. **(E)** AO staining in T24 cells. **(F)** AO staining in UMUC3 cells. *p<0.05, **p<0.01, ***p<0.001 and ****p<0.0001, vs. WT group.

In this study, the effect of cisplatin resistance on autophagy in BLCA. Thus, our experimental data presented higher expression patterns of LC3-II and lower expression patterns of p62 on cisplatin-resistant BLCA cells with comparing to wild-type cells, and the quantitative results also show the significant upregulation of LC3-II and p62 expression on T24R cell with comparing to wild type group. (Fig. 4A). Additionally, western blot analyses further confirm these findings also show higher protein levels of LC3-II and lower protein levels of p62 on resistance cells (Fig. 4B). Thus, we also analyzed ATG5 and ATG12 expressions which are important autophagy-related protein and markers, the results show that cisplatin-resistance increase the ATG5 and ATG12 protein expression on both cell lines (Fig. 4C-D). Additionally, acridine orange (AO) staining indicated distinct changes in the autophagic activity of resistant cells compared to their wild-type counterparts with significantly increasing the positive cells (Fig. 4E-F). These results underscore the pivotal role of autophagy and its associated proteins in mediating cisplatin resistance, offering insights into potential therapeutic targets for overcoming chemoresistance in BLCA.

### Effects of Autophagy inhibition on ABCC6 expression

**Figure 5:**
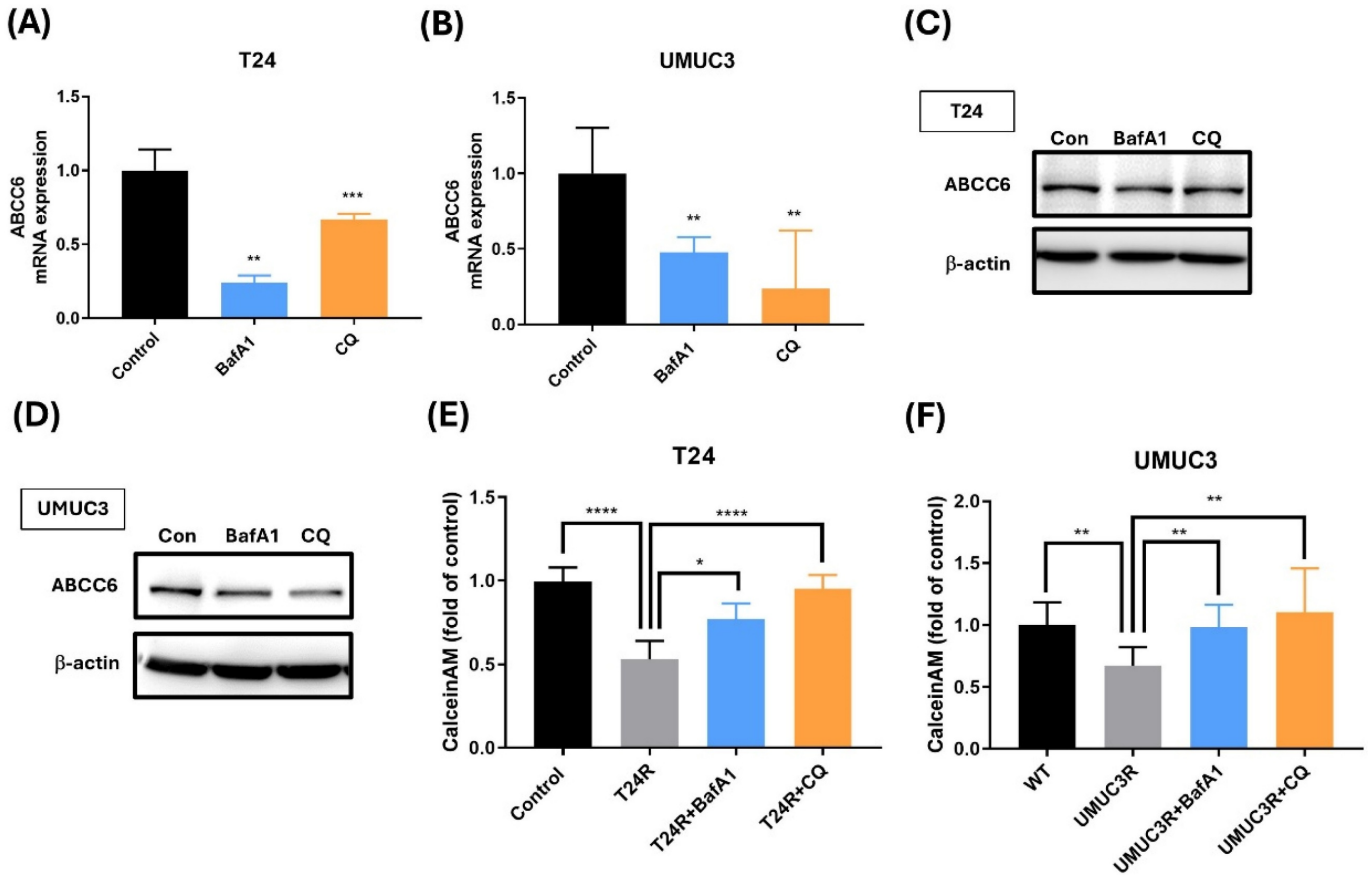
** The Role of ABCC6 in Drug Resistance of BLCA Cells. (A)** ABCC6 mRNA expression in T24 cells treated with BafA1 (bafilomycin A1) or CQ (chloroquine). **(B)** ABCC6 mRNA expression in UMUC3 cells treated with BafA1 or CQ. **(C)** ABCC6 protein levels in T24 cells are treated with BafA1 or CQ. **(D)** ABCC6 protein levels in UMUC3 cells treated with BafA1 or CQ. **(E)** Calcein AM accumulation in T24 cells with cisplatin resistance treated with BafA1 or CQ. **(F)** Calcein AM accumulation in UMUC3 cells with cisplatin resistance treated with BafA1 or CQ. *p<0.05, **p<0.01, ***p<0.001 and ****p<0.0001, vs. WT group.

To further confirm the connection between ABCC6 and autophagy, we also use the autophagy inhibitor on cisplatin-resistant BLCA cell. The experimental data presented in this image reveal significantly lower variations in ABCC6 mRNA expression after the BafA1 and CQ treatments on cisplatin-resistant T24 and UMUC3 cells (Fig. 5A-B). And similar results show the lower ABCC6 protein levels under BafA1 and CQ treatments of cisplatin-resistant T24 and UMUC3 cell lines (Fig. 5C-D). And the calcein AM accumulation shows the reversed effects of BafA1 and CQ treatment on cisplatin-resistant T24 and UMUC3 cell lines which downregulate the efflux of cisplatin and diminished intracellular drug accumulation functions (Fig. 5E-F). This visual representation underscores the pivotal role of autophagy in modulating drug resistance mechanisms, highlighting a potential therapeutic target for overcoming cisplatin resistance and improving treatment efficacy.

## Discussion

It is well known that drugs resistance is a significant challenge in the treatment of various cancers. In BLCA, drug resistance also is a significant barrier to effective treatment, especially cisplatin chemotherapy. Although, cisplatin is still important in the treatment of BLCA. Once BLCA progresses to the metastatic stage, only 35% of metastatic BLCA patients initially respond to cisplatin chemotherapy. Furthermore, the majority of BLCA patients who initially exhibit sensitivity to cisplatin eventually develop resistance 22. In this study, T24 and UMUC3 cell were used to develop the cisplatin resistance cell line, and in long-term survival ability of the model was assessed. Our results show that T24R cells are significantly increasing the cell viability compared to wild-type T24 cells. In previous study, the cell viability also increased after the cisplatin resistance was induced on T24 cell 23. Moreover, the colony formation assay shows that cisplatin resistance on T24 cell also increased the cell proliferation. And the Cis/UMUC3R also show similar results which the long-term survival ability and cell proliferation were increasing. Thus, cisplatin resistance not only increases the cell viability, but also promotes cell proliferation on BLCA.

Drug efflux transporters, such as P-glycoprotein (P-gp) and similar proteins, play a pivotal role in chemotherapy failure by actively removing anticancer drugs from cells. Among these, ATP-binding cassette (ABC) transporters constitute a large family of membrane proteins that harness energy from ATP hydrolysis to transport a diverse range of substrates across cellular membranes 13. These transporters are vital for various physiological functions, including lipid transport, nutrient absorption, and xenobiotic detoxification 24. However, they are also closely associated with multidrug resistance (MDR) in cancer, as they actively expel chemotherapeutic agents, thereby lowering their intracellular concentrations and diminishing their therapeutic efficacy 15. Thus, we exam the expression of different types of ABC transporters, including ABCC1, ABCC6, ABCC10, ABCB1 and ABCG2. The ABCC6 both show the higher expression on T24/R and UMUC3/R cells, and the level of calcein AM staining on cell also shows lower expression on Cis/T24R and Cis/UMUC3R cells. Then, the ABCC6 shRNA treatment on T24/R and UMUC3/R cell demonstrated that the ABCC6 inhibition affects the level of calcein AM staining on cell. Thus, we concluded that ABCC6 transporter may play an important role of cisplatin resistance on BLCA.

Moreover, much research indicated that drug resistance can induce autophagy as a cellular response to stress 25-27. Researchers are also investigating ways to target autophagy as part of cancer treatment to overcome drug resistance 28. And there is growing evidence that ABC transporters are also linked to autophagy, which is a cellular process that helps cells deal with stress, maintain homeostasis, and survive under adverse condition of drug treatment 29,30. Autophagy plays a crucial role in several cancer hallmarks, including cell survival, programmed cell death, metabolic deregulation, immune response modulation, the epithelial to-mesenchymal transition (EMT) process, cancer stem cell (CSC) enhancement, and the development of multidrug resistance (MDR) 30. As the previous studies demonstrated that cisplatin resistance can increase the Light chain 3-II (LC3-II) and autophagy protein 5 (ATG5) protein expression 31,32. Thus, we believe our findings contribute novel insights into the role of ABCC6 in BLCA chemoresistance. Unlike the well-studied ABCC1/2 transporters, ABCC6 has not been previously linked to autophagy-mediated drug resistance in BLCA. By revealing this novel axis, our study opens the door to exploring autophagy modulation as a viable co-treatment strategy in cisplatin-refractory cases.

Our previous study indicated that target to autophagy with influencing the related marker, such as LC3-II, ATG5, ATG12 and p62 protein expression can improve the progression of BLCA via regulating micro-RNA expression of BLAC, such as hsa-miR-30a-3p or hsa-miR-34 33,34 or the miconazole. In this study, we preliminary evaluate LC3-II and ATG5 on T24R and UMUC3R cells, the result show that cisplatin also increasing the LC3-II and ATG5. And the autophagy related 12 also increasing on T24R and UMUC3R cells with p62 protein decreasing on T24R cells. And AO staining also indicates that autophagy function was increasing on T24R and UMUC3R cells. And the inhibition of autophagy is also an effective strategy for drug resistance on the treatment cancer 27,35. Our previous studies also indicated that blocking the autophagy on BLCA by autophagy blockade, such as the BafA1 and CQ, can inducing programmed death ligand-1 (PD-L1) up-regulation with inhibiting miR-34a expression on BLCA. In this study we found that autophagy BafA1 and CQ, were used to exam the correction of cisplatin resistance associated with autophagy on BLCA. And the ABCC6 expression was reduced with the calein AM staining showing the reverse effect of cisplatin resistance after BafA1 or CQ treatment on T24R and UMUC3R cells. Thus, we concluded that cisplatin resistance can be reversed by the inhibition of autophagy. And the strong association of ABCC6 with cisplatin resistance highlights its potential as a biomarker for chemoresistance in BLCA patients. Since autophagy inhibitors like CQ are already U.S. Food and Drug Administration (FDA)-approved, they may be repurposed in combination therapies with cisplatin. Ongoing efforts to validate ABCC6 expression in clinical samples will help assess its predictive value. Our work sets the stage for clinical trials incorporating autophagy modulation in treatment-resistant BLCA. However, the role of PD-L1/ miR-34a axis on cisplatin inducing still need to discussion for future work. And for future investigation, we also will design to exam the relationship between micro-RNA and cisplatin resistance with could improving cisplatin inducing resistance on BLAC cell. Given the clinical significance of drug and platinum resistance in BLCA, we will aim to investigate the expression profiles of ABCC6 and autophagy-related markers in clinical BLCA specimens. To further elucidate the mechanisms underlying cisplatin resistance, we will employ an *in vivo* xenograft model to evaluate the therapeutic potential of ABCC6-specific inhibitors and autophagy modulators, with the goal of identifying candidates for clinical applications. Additionally, we will extend our investigation to other urological malignancies to explore the broader relevance of platinum resistance mechanisms. To uncover novel pathways and genetic contributors associated with cisplatin resistance, mRNA sequencing will be performed, enabling the identification of differentially expressed genes and signaling cascades that may be modulated by platinum-resistance.

## Conclusion

**Figure 6:**
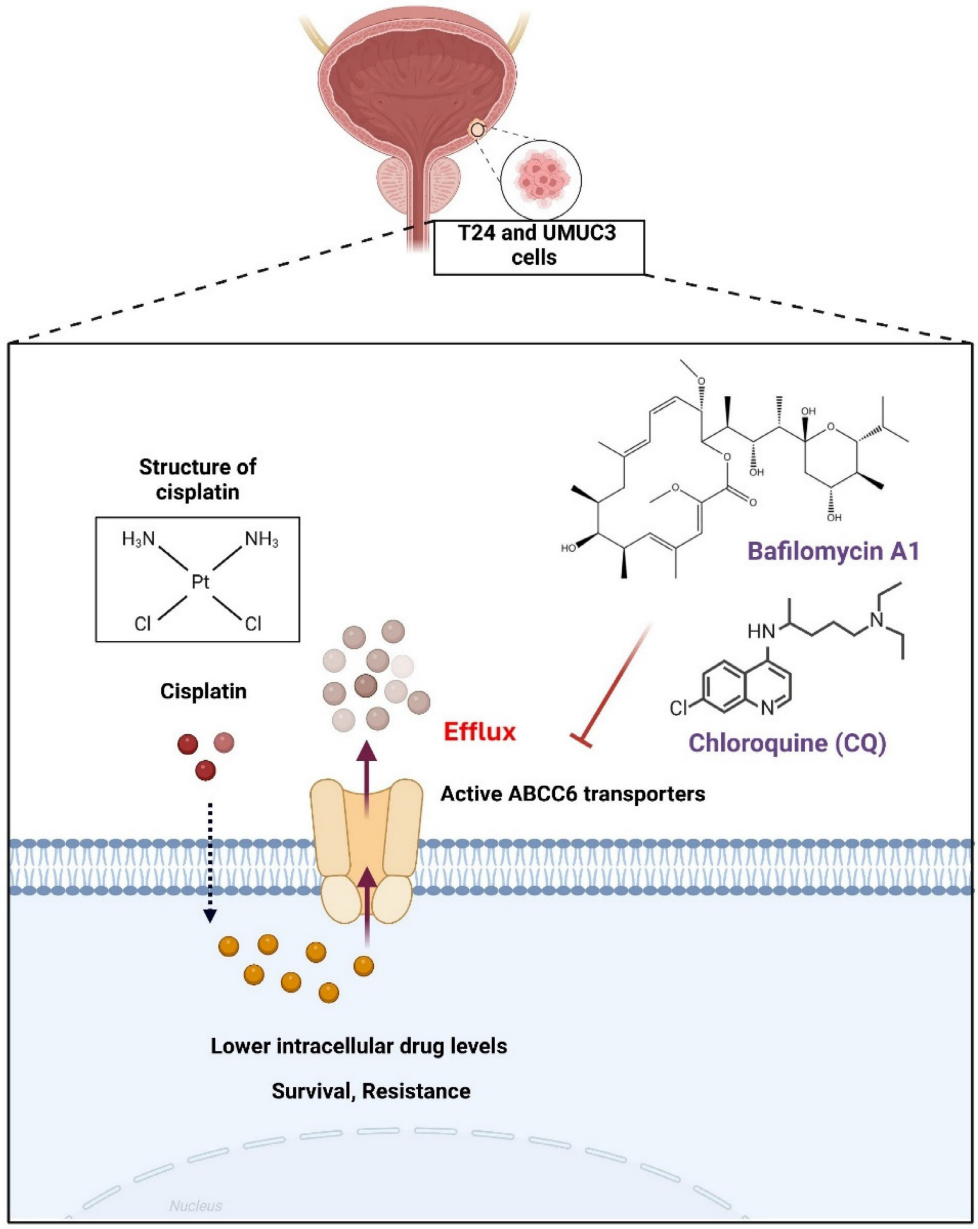
** Mechanism of Cisplatin Resistance Mediated by ABCC6 Transporters.** This illustration highlights a crucial mechanism in chemotherapy resistance, emphasizing the role of ABCC6 transporters in reducing the intracellular concentration of cisplatin, thereby enhancing cell survival and resistance.

In conclusion, we determined that cisplatin could promote ABCC6 transporter expression to contribute the cisplatin resistance on BLCA (Fig. 6), and the inhibition of autophagy show as the potential strategy for treating drug resistant on BLCA.

## Figures and Tables

**Table 1:** Primer sequences for qPCR

Forward primers (5' to 3')	Reverse primers (5' to 3')
AGGACACGTCGGAACAAGTC	GGAAGTAGGGCCCAAAGGTC
CACAGTTTGTGCTGTCCTGC	CCAAGCGACCAGAGGTCTTT
CCTAGTGCTGACCGTGTTGT	TAGGTTGGCTGCAGTCTGTG
CGTGGTTGGAAGCTAACCCT	TGCTGCCAAGACCTCTTCAG
TGATAAATGGAGCACCGCGA	GCCAGTTGTAGGCTCATCCA
CTGGGCTACACTGAGCACC	AAGTGGTCGTTGAGGGCAATG
